# WT1 peptide analogue WT1-126Y enhances leukemia lysis

**DOI:** 10.1186/2051-1426-2-S1-O3

**Published:** 2014-02-24

**Authors:** Ghofran Alqudaihi, Cynthia Lehe, Monther Al-Alwan, Hazem Ghebeh, Anne Dickinson, Said Dermime

**Affiliations:** 1 King Faisal Specialist Hospital & Research center, Riyadh, Saudi Arabia; 2 Dasman Diabetes Institute, Kuwait; 3 University of Newcastle upon Tyne, Newcastle Upon Tyne, UK; 4 King Fahad Specialist Hospital, Dammam, Saudi Arabia

## Background

The Wilms Tumour Antigen 1 (WT1) has been shown to be expressed at low levels in some normal cells. Therefore, many of the potential CTL epitopes against this antigen may be absent or suboptimal. To this end, different groups introduced modifications in the sequence of the anchor positions of these “sub-optimal” peptides to improve their binding to HLA class I molecules and to increase their immunogenicity.

## Aim

To explore the feasibility of using an approach that enhances the immunogenicity of low-avidity restricted peptides without altering their antigenic specificity. This approach consists of replacing the first amino acid of two known HLA-A0201-restricted WT1-derived peptides (Db126 and WH187) by a tyrosine (Y).

## Findings

The modified WT1-Db126 showed enhanced binding ability to the HLA-A*0201 molecule, increased the frequency of IFN-γ producing cytotoxic T lymphocyte (CTL) and boosted the lytic activity of the generated CTL against HLA-matched leukaemia cells. Interestingly, the CTL line generated with the modified epitope was able to recognize the wild-type peptide presented by target cells.

## Conclusions

This study provide evidence that peptide modification results in a better immune response against cancer and further support the use of this strategy as a potential approach for the development of a leukemia-vaccine.

